# West Texas Medical Society

**Published:** 1890-10

**Authors:** G. W. Kerr, D. Berrey

**Affiliations:** President; San Antonio, Texas; Secretary; San Antonio, Texas


### WEST TEXAS MEDICAL SOCIETY.

SAN ANTONIO, TEXAS, October 1st, 1890.

DEAR DOCTOR:—You are cordially invited to attend the Sixth Quarterly and regular Annual Meeting of the West Texas Medical Association, to be held in San Antonio, October 29th, 1890, at Scholz's Hall. Three meetings will be held daily; at 10 a. m., 3 p. m. and 8 p. m.

Papers will be presented by the following gentlemen: Some Reflections and the Cause and Treatment of Potts Disease of the Spine, by Jos. L. Bauer, M. D., of St. Louis, Mo. Inflammation, Ulceration and Perforation of the Appendix Vermiformis, by A. S. McDaniel, of San Antonio. Peroxide of Hydrogen, by G. W. Kerr, M. D., of Waelder. Report of Cases of Perineal Section, by B. F. Ringsley, M. D., of San Antonio. Report of Case of Aneurism of the Palmar Arch, by R. Menger, M. D., of San Antonio. Hay Fever, by H. J. Trolinger, M. D., of San Antonio. Other papers of interest by local members have been promised.

At the night session the election of officers to serve for the ensuing year will be held. The session will close with the regular annual banquet. Very respectfully,

G. W. KERR, M. D., President.

D. BERREY, M. D., Secretary.

